# Mas receptor mediates cardioprotection of angiotensin‐(1‐7) against Angiotensin II‐induced cardiomyocyte autophagy and cardiac remodelling through inhibition of oxidative stress

**DOI:** 10.1111/jcmm.12687

**Published:** 2015-10-30

**Authors:** Li Lin, Xuebo Liu, Jianfeng Xu, Liqing Weng, Jun Ren, Junbo Ge, Yunzeng Zou

**Affiliations:** ^1^ Department of Cardiovascular MedicineEast HospitalTongji University School of MedicineShanghaiChina; ^2^ Shanghai Institute of Cardiovascular DiseasesZhongshan Hospital and Institute of Biomedical ScienceFudan UniversityShanghaiChina

**Keywords:** autophagy, heart failure, angiotensin‐(1‐7), oxidative stress, Mas

## Abstract

Angiotensin II (Ang II) plays an important role in the onset and development of cardiac remodelling associated with changes of autophagy. Angiotensin1‐7 [Ang‐(1‐7)] is a newly established bioactive peptide of renin–angiotensin system, which has been shown to counteract the deleterious effects of Ang II. However, the precise impact of Ang‐(1‐7) on Ang II‐induced cardiomyocyte autophagy remained essentially elusive. The aim of the present study was to examine if Ang‐(1‐7) inhibits Ang II‐induced autophagy and the underlying mechanism involved. Cultured neonatal rat cardiomyocytes were exposed to Ang II for 48 hrs while mice were infused with Ang II for 4 weeks to induce models of cardiac hypertrophy *in vitro* and *in vivo*. LC3b‐II and p62, markers of autophagy, expression were significantly elevated in cardiomyocytes, suggesting the presence of autophagy accompanying cardiac hypertrophy in response to Ang II treatment. Besides, Ang II induced oxidative stress, manifesting as an increase in malondialdehyde production and a decrease in superoxide dismutase activity. Ang‐(1‐7) significantly retarded hypertrophy, autophagy and oxidative stress in the heart. Furthermore, a role of Mas receptor in Ang‐(1‐7)‐mediated action was assessed using A779 peptide, a selective Mas receptor antagonist. The beneficial responses of Ang‐(1‐7) on cardiac remodelling, autophagy and oxidative stress were mitigated by A779. Taken together, these result indicated that Mas receptor mediates cardioprotection of angiotensin‐(1‐7) against Ang II‐induced cardiomyocyte autophagy and cardiac remodelling through inhibition of oxidative stress.

## Introduction

The renin–angiotensin system (RAS) is a cascade of essential regulators for a wide variety of physiological and pathophysiological events in the cardiovascular system. Overactivation of the angiotensin‐converting enzyme (ACE)–angiotensin II (Ang II)–angiotensin II type 1 receptor (AT1R) axis, the classical pathway of RAS, has been found to possess a pivotal role in the aetiology of heart failure 1, 2. Recently, accumulating evidence has suggested a close tie between RAS overactivation and autophagy induction 3, 4, 5. Porrello *et al*. provided evidence for the first time demonstrating the interplay between Ang II and autophagy through Ang II–AT1R coupling in neonatal cardiomyocytes 6. Subsequent work showed that Ang II promoted autophagic activity in podocytes, leading to renal injury 7.

Angiotensin1‐7 [Ang‐(1‐7)] is a newly established bioactive peptide of RAS, which is formed from Ang II by ACE2 and has been shown to oppose the deleterious effects exerted by the ACE–Ang II –AT1R axis 8. Under pathological conditions, it has been recognized that Ang‐(1‐7) may opposes Ang II‐exerted cardiac responses through binding to the Mas receptor to elicit a cascade of signalling pathways leading to vasodilation and anti‐hypertrophic actions 9, 10. Although Ang II plays a critical role in cardiomyocyte autophagy, the importance of the Ang‐(1‐7)/Mas receptor axis in this context remains essentially elusive. Therefore, this study was designed to determine whether Ang‐(1‐7) is effective in ameliorating the unfavourable effects of Ang II on cardiomyocyte autophagy and the underlying mechanism of action involved with a focus on the Mas receptor using the selective Mas receptor antagonist A779 11.

## Materials and methods

### Animal models

All animal procedures were approved by the Animal Care and Use Committee of Fudan University and were in compliance with the Guidelines for the Care and Use of Laboratory Animals published by the National Academy Press (NIH Publication No. 85‐23, revised in 1996). In brief, C57BL/6 male mice, aged 8–10 weeks, were purchased from the Jackson Laboratory (Bar Harbor, ME, USA). Ang II (1000 ng/kg/min.; Sigma‐Aldrich, St. Louis, MO, USA), Ang‐(1‐7) (500 μg/kg/day; Sigma‐Aldrich) and A779 (500 μg/kg/day; Sigma‐Aldrich) were continuously administered using Alzet osmotic minipumps (Model 2002; DURECT, Cupertino, CA, USA) implanted subcutaneously into the experimental animals. Four weeks later, all mice were killed and hearts were excised for further examination.

### Echocardiography and haemodynamic measurements

Transthoracic echocardiography was performed with 30 MHz high frequency scanhead (VisualSonics Vevo770; VisualSonics Inc., Toronto, ON, Canada) 12. All measurements, averaged for five consecutive cardiac cycles, were carried out by three experienced technicians unaware of experimental group identity 13. Blood pressure (BP) was evaluated as described 14, 15. A micronanometer catheter (Millar 1.4F, SPR 835; Millar Instruments, Inc., Houston, TX, USA) was inserted into the right common carotid artery, while the transducer was connected to a Power Laboratory system (AD Instruments, Castle Hill, NSW, Australia) to record BP readings.

### Morphology and histological analyses

Excised hearts were weighed, perfused with PBS and fixed with 4% polyformaldehyde for global morphometry and then with 10% formalin for further histological analysis. The papillary muscle levels were used for heart excision for LV characterization of cardiomyocyte hypertrophy. We took serial cuts to evaluate cardiomyocyte hypertrophy. Paraffin‐embedded hearts were sectioned at 4‐μm thickness and stained with haematoxylin and eosin. Cardiomyocyte morphology and histology were visualized under a high magnification to assess cross‐sectional area (CSA) using a video camera (Leica Qwin 3, Leica Microsystems, Wetzlar, Germany) attached to a micrometer. Twenty randomly chosen fields were evaluated from each cross‐section of the LV free wall.

### Cell culture and treatment

Neonatal cardiomyocytes were prepared from 1 to 3 days Sprague–Dawley rats by trypsin digestion method as described elsewhere. Briefly, neonatal rat ventricles were minced into pieces and subjected to 0.125% trypsin digestion in hank's balanced salt solution. After 2 hrs of cell attachment, cardiomyocytes were collected and maintained for 24 hrs in DMEM/F12 containing 10% FBS with antibiotics. Then serum was deprived for 24 hrs prior to experiments. After pretreated with Ang‐(1‐7) (10^−6^ mol/l) or A779 (10^−6^ mol/l) for 30 min., AngII (10^−6^ mol/l) or vehicle was added to the cells. After 48 hrs incubation, cardiomyocytes were collected and lysed for the further analysis.

### [^3^H] leucine incorporation

The cultured cardiomyocytes were incubated with [^3^H] leucine (1 μCi/ml) in the plates, and then imposed with a 48‐hour AngII treatment. Afterwards, they were treated with 5% trichloroacetic acid, and the protein precipitates were dissolved in 1 ml of 100 mmol/l NaOH. The radio activities were determined with a liquid scintillation counter.

### Real‐time RT‐PCR

Total RNA was isolated from LV tissues or culture cells using TRIZol^®^ reagent according to the manufacturer's instruction. After purification, RNA was subjected to real‐time RT‐PCR analysis for the expression of atrial natriuretic peptide (*Anp*) and skeletal α‐actin (*Saa*) on a Bio‐Rad IQ5 multicolour detection system (Hercules, CA, USA). The melting curves and quantization were analysed using Light Cycler and Rel Quant software, respectively. A comparative CT method was used to determine relative quantification of RNA expression 16. All PCR reactions were performed at least in triplicate.

### Western blot analysis

The homogenized ventricles tissue or cultured cells were incubated in lysis buffer (50 mM Tris‐HCl, pH 7.4; 150 mM NaCl; 5 mM ethylenediaminetetraacetic acid; 25 mM NaF; 1% Triton‐X 100; 1% NP‐40; 0.1 mM Na3VO4; 12.5 mM b‐glycerophosphate; 1 mM phenylmethanesulfonylfluoride (PMSF) and complete protease inhibitor cocktail). A total of 80 ml of lysis buffer was used in the particulate fractions separation in WB. The lysates were clarified at 13,000 r.p.m. for 10 min., and the supernatants were quantified and subjected to SDS‐PAGE and western blotting on Immobilon‐P membranes (Millipore Corporation, Billerica, Massachusetts). The blotted membranes were incubated with antibodies against LC3b‐II or P62 (Cell Signaling Technology Inc., Beverly, MA, USA), and subjected to an ECL Detection system (GE Healthcare, Little Chalfont, Buckinghamshire, United Kingdom).

### Autophagic flux assessment

For autophagic flux assessment, cardiomyocytes were treated with vehicle or chloroquine (CQ, 20 μmol/l) 17 for 4 hrs and cell lysates were prepared for the detection of LC3b‐II protein abundance.

### Generation of GFP‐LC3 vector and fluorescent microscopic analysis

A lentiviral vector containing GFP‐LC3 reporter (GFP‐LC3) was constructed 18. Cardiomyocytes were transfected with lentivirus particles (MOI = 20) and then were treated with AngII for 48 hrs. Cells were visualized under a fluorescent microscope 19.

### Immunofluorescence

Following cell culture on silicon‐based plates in serum‐free DMEM for 48 hrs, cardiomyocytes were incubated with anti‐α‐MHC (catalogue 05‐716, Upstate, Millipore Corporation, Billerica, Massachusetts, USA) and LC3b‐II (catalogue 2775; Cell Signaling Technology Inc.). Samples were then incubated with secondary antibodies conjugated with FITC or Alex (catalogue A21206; Invitrogen, Carlsbad, CA, USA) according to the manufacturer's instructions. The surface areas of cardiomyocytes and LC3b‐II were determined using an image analysis software (Leica Qwin 3) and were calculated using the mean of 100 to 120 cells from randomly selected fields.

### Determination of oxidative stress

Malondialdehyde (MDA) content and superoxide dismutase (SOD) activity were used as indices to assess oxidative stress. Following the indicated treatments, the LV was collected and homogenated. Measurement was performed according to the manufacturer's instructions (Jiancheng Bioengineering Institute, Nanjing, China) using a microplate reader (Molecular Devices, Sunnyvale, CA, USA).

### Statistical analysis

Data are shown as means ± S.E.M. Comparison was performed by one‐way anova followed by Newman–Keuls test for *post hoc* analysis to determine the difference among the groups.

## Results

### Ang‐(1‐7) inhibited Ang II‐induced autophagy in cultured cardiomyocytes

**Figure 1:**
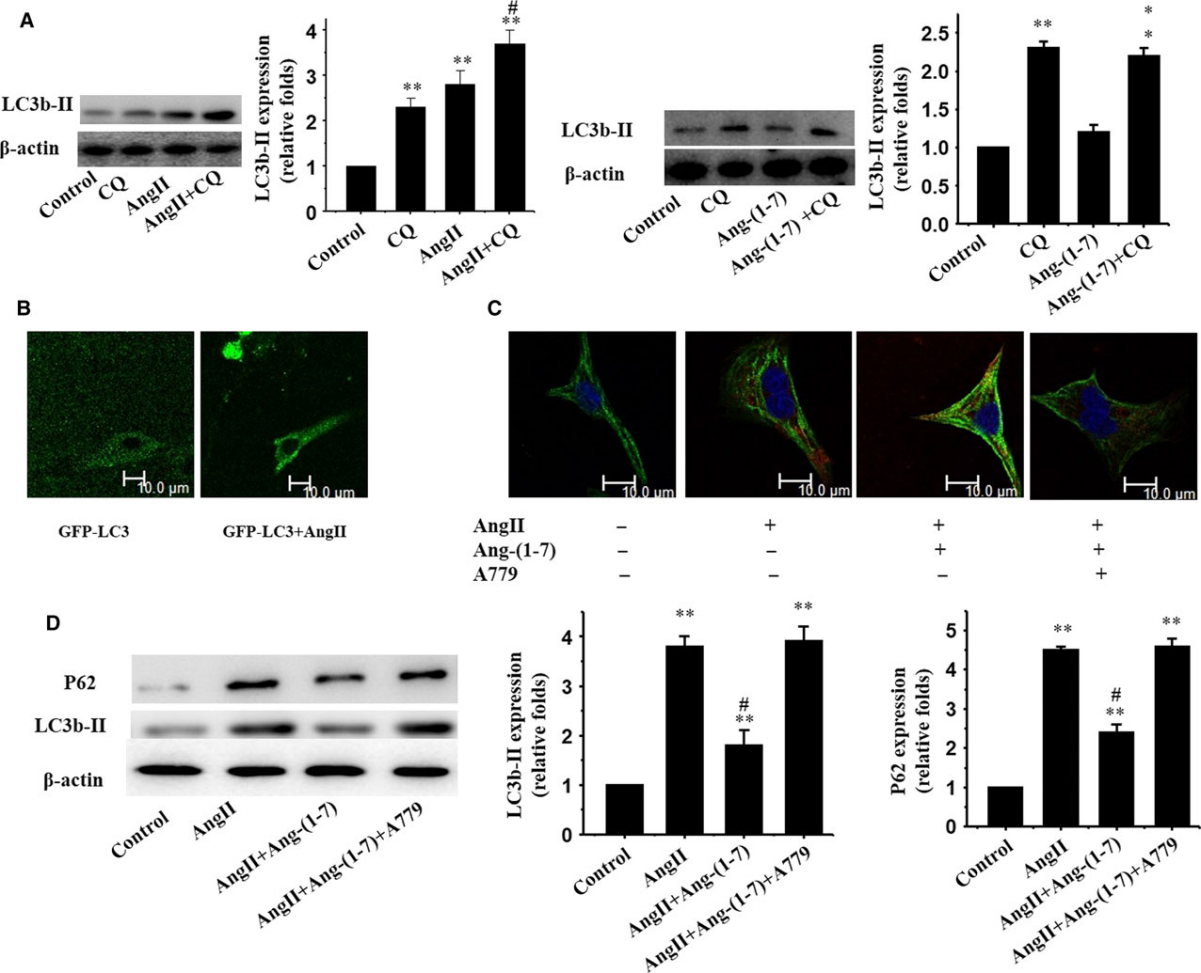
Ang‐(1‐7) inhibits Ang II‐induced autophagy *via* the Mas receptor in cultured cardiomyocytes. (**A**) Western blot analysis for LC3b‐II using the anti LC3b‐II antibody; β‐actin was employed as the loading control; (**B**) Fluorescent microscopic analysis for GFP‐LC3; (**C**) Immunofluorescent staining for LC3b‐II (red) and α‐MHC (green); (**D**) Western blot analysis for expression of LC3b‐II and P62; β‐actin was used as the loading control. Representative photograms from three independent experiments are shown. All data are expressed as mean ± S.E.M. from three independent experiments, **P < 0.01 *versus* cardiomyocytes in the control; #*P* < 0.05 *versus* cardiomyocytes with Ang II.

We examined autophagy in cardiomyocytes following Ang II treatment. Cultured cardiomyocytes exhibit a significant rise in cardiomyocyte autophagy as demonstrated by increased LC3b‐II following a 48‐hour Ang II treatment (Fig. 1A). To examine whether AngII affects autophagic flux, cardiomyocytes were subjected to incubation of AngII in the absence and presence of chloroquine, to inhibit lysosomal acidification and autophagosome–lysosome fusion. The control group demonstrated a basal level of cardiomyocyte autophagy, with autophagosome accumulation (2.3‐fold increase) in the presence of chloroquine, suggesting the intact autophagic flux. AngII treatment elicited a 3.7‐fold rise in autophagosome abundance compared with control group, implying the induction of autophagosome formation (Fig. 1A). However, Ang‐(1‐7) treatment failed to affect autophagic flux (Fig. 1A). To discern the characteristics of autophagy elicited by AngII, the GFP‐LC3 puncta depicting accumulation of autophagosomes in cardiomyocytes were visualized using a fluorescence microscope 20. Fluorescent microscopic analysis displayed the unique punctate pattern of LC3 in AngII‐treated cardiomyocytes, distinct from the diffused distribution pattern of LC3 found in control cardiomyocytes (Fig. 1B). Interestingly, treatment of Ang II‐stimulated cardiomyocytes with Ang‐(1‐7) overtly suppressed Ang II‐induced autophagy (Fig. 1C and D).

### Ang‐(1‐7) inhibited Ang II‐induced hypertrophic responses in cultured cardiomyocytes

**Figure 2:**
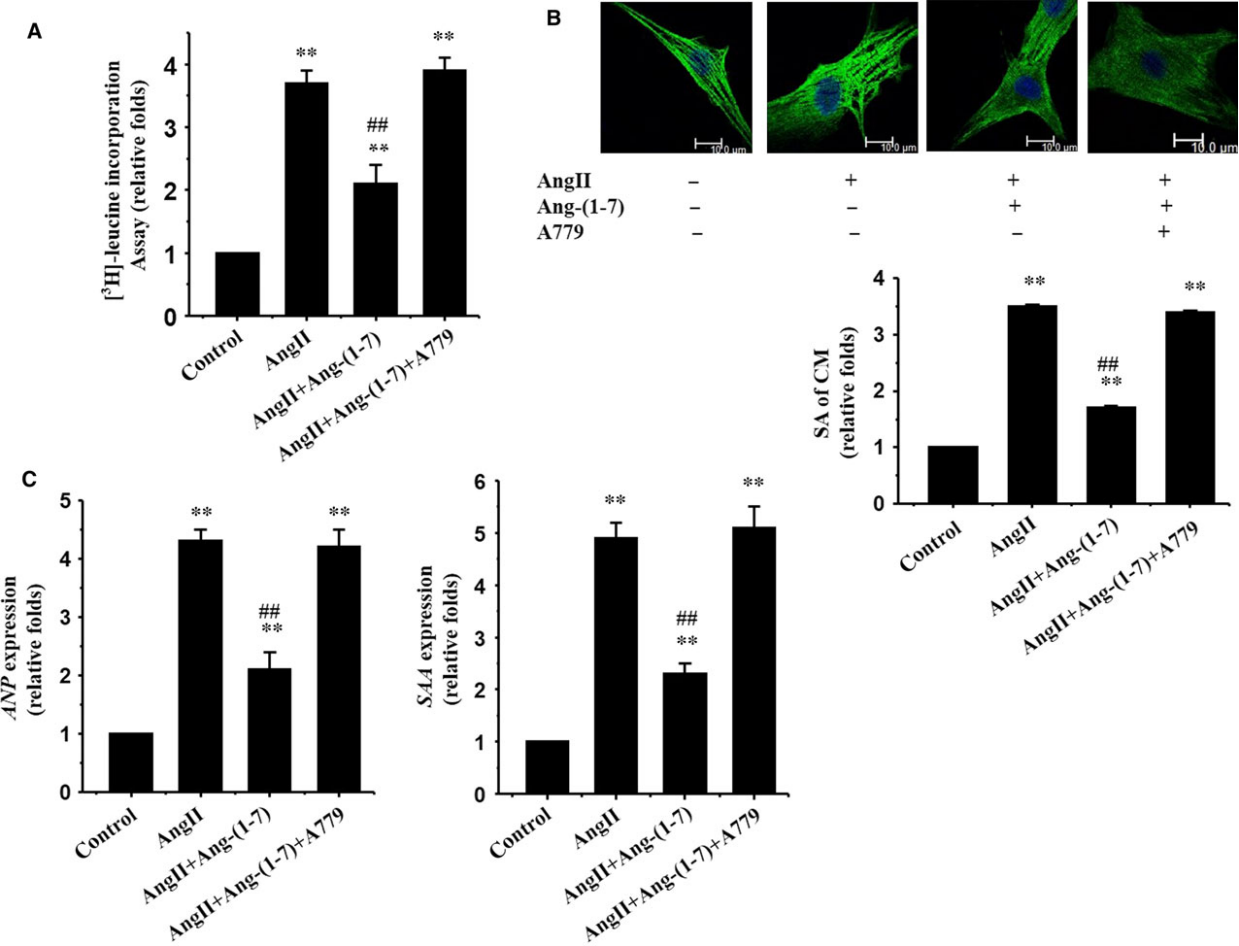
Ang‐(1‐7) inhibits Ang II‐induced cardiomyocyte hypertrophy *via* a Mas receptor‐dependent manner in cultured cardiomyocytes. Cultured cardiomyocytes of neonatal rats treated by vehicle (Control) or Ang II in the absence or presence of Ang‐(1‐7) (10^−6^ mol/l) or A779 (10^−6^ mol/l). (**A**) [^3^H]‐Leucine incorporation in cardiomyocytes; (**B**) Cardiomyocyte morphology and size; cardiomyocytes were subjected to immunofluorescent staining for α‐MHC (green) and DAPI; Representative photographs were shown from three independent experiments (scale bar: 10 μm); surface area (SA) of cardiomyocytes was evaluated by measuring 100 cardiomyocytes from each dish; (**C**) Expressions of *Anp* and *Saa* genes evaluated by real‐time RT‐PCR; β*‐Actin* was used as the internal control. All data are expressed as mean ± S.E.M. from three independent experiments, **P < 0.01 *versus* cardiomyocytes in the control; ##*P* < 0.01 *versus* cardiomyocytes with AngII.

The cultured neonatal rat cardiomyocytes were subjected to test the rate of the cell protein synthesis. Ang II significantly increased the protein synthesis of cardiomyocytes (Fig. 2A). We then measured the SA of cardiomyocytes by immunostaining using antibody against α‐MHC. Similarly, treatment with Ang II significantly increased the SA of cardiomyocytes (Fig. 2B). Similar results were observed in detection of expression of foetal type genes, *Anp* and *Saa*, both of which were up‐regulated in AngII‐treated cardiomyocytes (Fig. 2C). Interestingly, the Ang II‐induced hypertrophic responses were significantly attenuated by treatment with Ang‐(1‐7) (Fig. 2A–C).

### Ang‐(1‐7) inhibited Ang II‐induced autophagy and myocardial fibrosis *in vivo*

**Figure 3:**
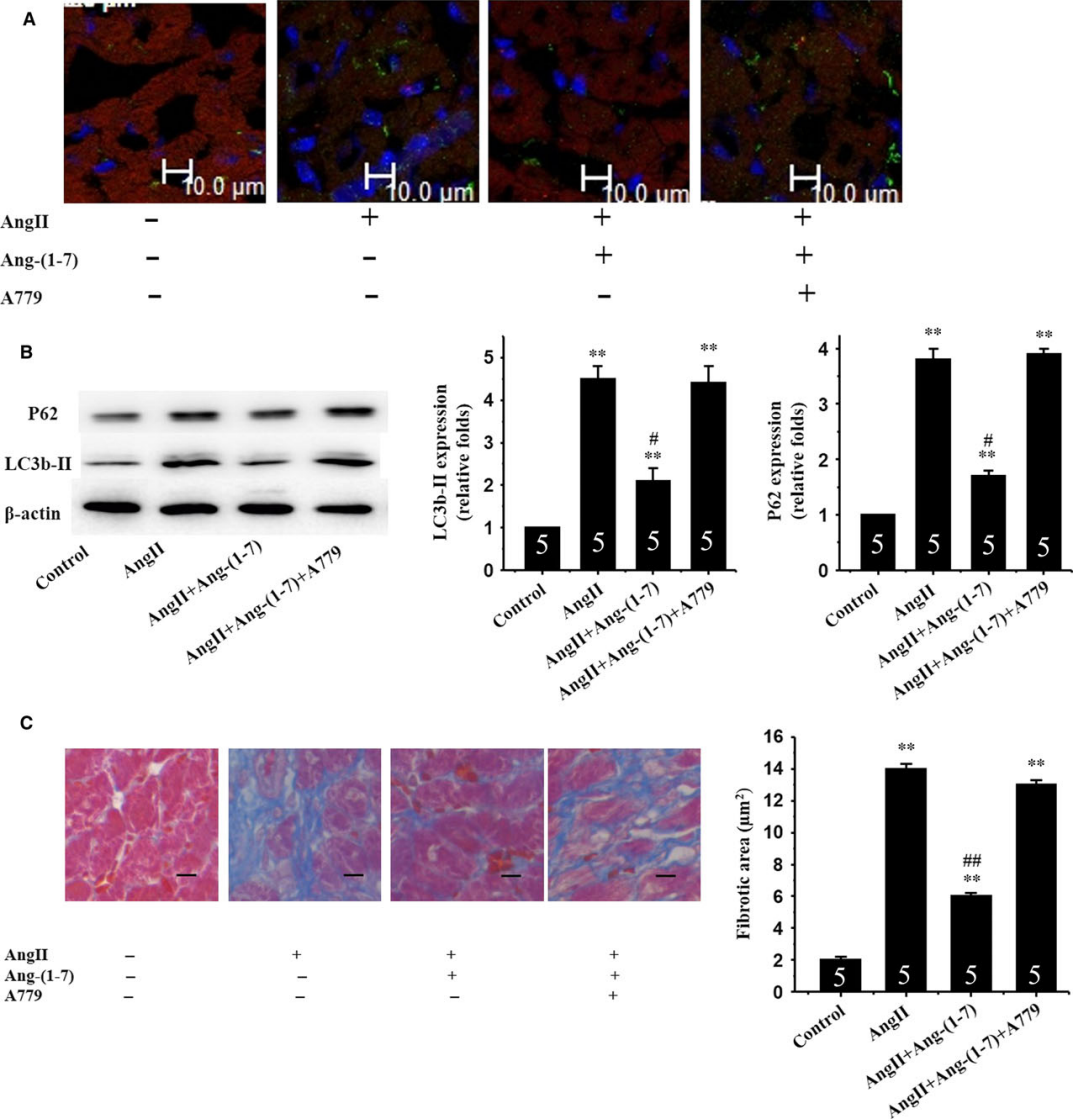
Ang‐(1‐7) inhibits AngII‐induced autophagy and myocardial fibrosis *via* a Mas receptor‐dependent mechanism in mice. (**A**) Immunofluorescent staining for expression of LC3b‐II proteins using an anti LC3b‐II antibody (green); (**B**) Western blot analysis for expression of LC3b‐II and P62; β‐actin in whole cell lysate used as the loading control; (**C**) Myocardial interstitial fibrosis. Representative Masson trichrome‐stained LV areas are shown. Blue areas indicate fibrotic staining. Fibrosis was measured in whole LV section (five sections for each mouse heart). Insets: Representative micrographs from five independent experiments. **p<0.01 vs. in the control; #p< 0.05, ##p< 0.01 vs. in the AngII‐treated mice.

Extensive cardiomyocyte autophagy and myocardial fibrosis have been speculated to play a role in the transition from adaptive hypertrophy to heart failure 21, 22. To this end, the effect of Ang‐(1‐7) on myocardial autophagy and interstitial fibrosis was evaluated in the face of Ang II exposure. Sustained AngII treatment triggered a progressive cardiomyocyte autophagy (Fig. 3A and B) and interstitial fibrosis in mice (Fig. 3C), the effect of which was significantly attenuated by Ang‐(1‐7) treatment (Fig. 3A–C).

### Ang‐(1‐7) reduced cardiac remodelling and preserved cardiac function following Ang II treatment

**Figure 4:**
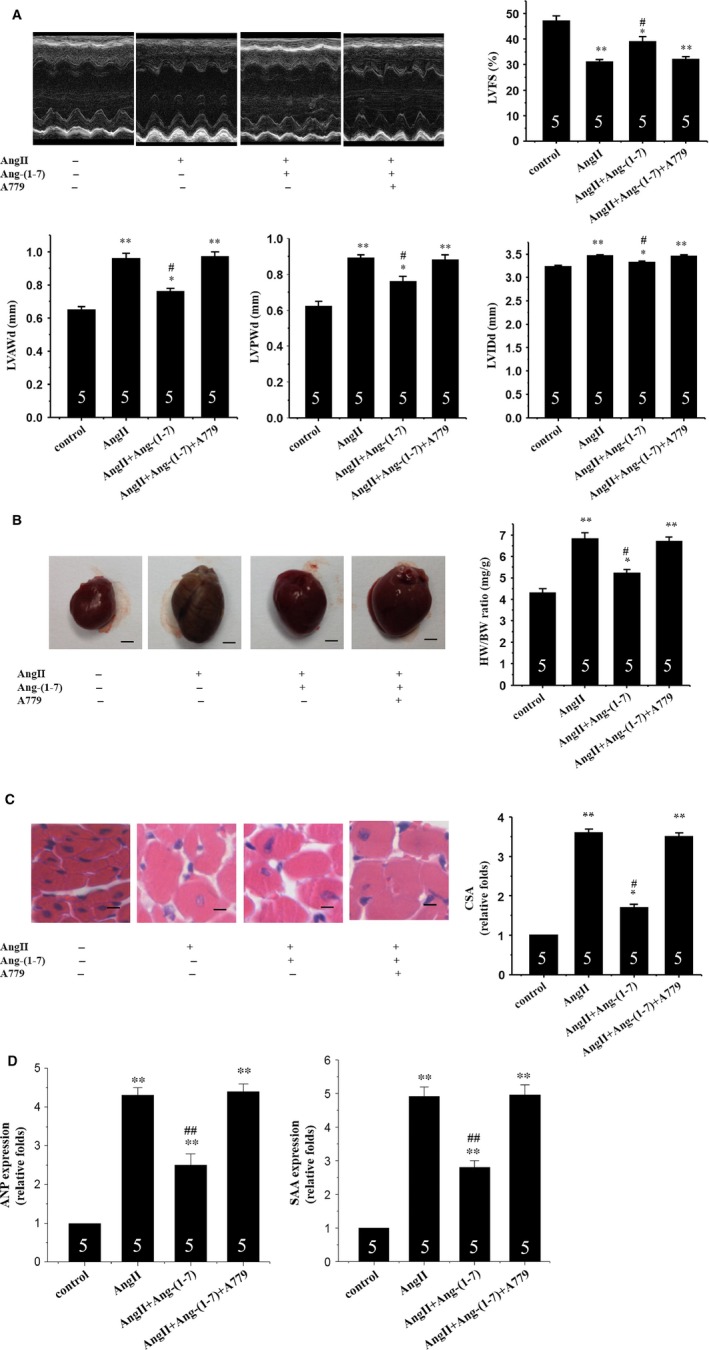
Ang‐(1‐7) inhibits AngII‐induced cardiac remodelling *via* a Mas receptor‐dependent mechanism in mice. (**A**) Echocardiographic analysis with representative M‐mode tracings from five mice. All echocardiographic data are shown as mean ± S.E.M. from five mice; LVAWd, LV anterior wall thickness at end‐diastole; LVPWd, LV posterior wall thickness at end‐diastole; LVIDd, LV internal dimension at end‐diastole; LVFS, LV fraction shortening; (**B**) Heart morphology and weight; representative global heart photographs of five mice (scale bar: 2 mm); heart weight to bodyweight ratio (HW/BW) measured from five mice; (**C**) Haematoxylin and eosin‐stained LV sections of mice; scale bar: 20 μm; cross‐sectional area (CSA) of cardiomyocyte measured from five sections for one heart and five hearts examined; (**D**) Expression of *Anp* and *Saa* genes evaluated by the real‐time RT‐PCR. β*‐Actin* used as internal control; Representative photograms from five hearts. All values are expressed as mean ± S.E.M. of five mice in all groups; *P < 0.05, **P < 0.01 *versus* in the control; #*P* < 0.05, ##*P* < 0.01 *versus* in the AngII‐treated mice.

To investigate whether Ang‐(1‐7) reduces cardiac remodelling *in vivo*, we treated the mice with Ang II. Four weeks later, echocardiographic measurement showed that the AngII‐infused mice developed a significant cardiac remodelling including the increased LV anterior wall at the end‐diastole, LV posterior wall at the end‐diastole, LV internal dimension at end‐diastole and decreased LV fractional shortening (Fig. 4A). Gross heart size and heart weight to bodyweight ratio (HW/BW) were also increased by treatment with Ang II (Fig. 4B). Measurement of CSA of cardiomyocytes in haematoxylin and eosin‐stained LV sections revealed that Ang II significantly enlarged the cardiomyocytes size (Fig. 4C). Up‐regulation of *Anp* and *Saa* was also observed in hearts from AngII‐treated mice (Fig. 4D). However, these maladaptive responses were significantly ameliorated in Ang‐(1‐7)‐treated mice (Fig. 4A–D). These results suggest that Ang‐(1‐7) is capable of preventing cardiac remodelling and contractile dysfunction resulted from a 4‐week period of AngII treatment.

### Effects of Mas receptor antagonism on Ang‐(1‐7)‐mediated actions in AngII‐treated cardiomyocytes

To determine if Ang‐(1‐7) exerts its actions through the Mas receptor, the selective Mas receptor antagonist A779 was used. Cardiomyocytes were exposed to Ang II with or without Ang‐(1‐7) and/or A779. Our data revealed that A779 prevented the beneficial effect of Ang‐(1‐7) on Ang II‐induced autophagy both *in vitro* (Fig. 1C and D) and *in vivo* (Fig. 3A and B).

### Effect of Ang(‐1‐7) on oxidative stress in the heart

**Figure 5:**
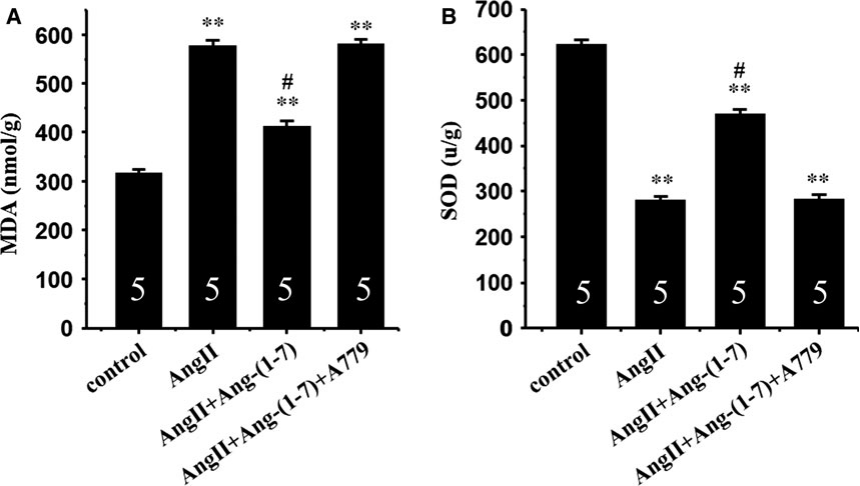
Ang‐(1‐7) inhibits AngII‐induced oxidative stress *via* a Mas receptor‐dependent mechanism in mice. (**A**) Malondialdehyde (MDA) level; (**B**) superoxide dismutase (SOD) activity. All values are expressed as mean ± S.E.M. of five mice in all groups; **P < 0.01 *versus* in the control; #*P* < 0.05 *versus* in AngII‐treated mice.

To investigate the effect of Ang‐(1‐7) on oxidative stress in mouse heart, the levels of MDA and SOD were evaluated. MDA is an indicator for lipid peroxidation to estimate the levels of oxidative stress. In Ang II‐treated mice, the MDA level was much higher than that of the control mice (Fig. 5A). Chronic infusion of Ang‐(1‐7) led to a significant decrease in MDA level (Fig. 5A). Co‐infusion with A‐779 abolished the Ang‐(1‐7)‐induced reduction in MDA levels (Fig. 5A).

Superoxide dismutase represents the first line of defence against oxidative stress, by catalysing the dismutation reaction of superoxide anion to the more stable hydrogen peroxide. Ang II treatment significantly decreased SOD activity, the effect of which was attenuated by chronic infusion of Ang‐(1‐7). Interestingly, Ang‐(1‐7)‐elicited beneficial effect against Ang II on SOD activity was nullified by A‐779 (Fig. 5B).

## Discussion

Autophagy, an evolutionarily conserved process of lysosome‐dependent turnover of damaged proteins and organelles 23, plays a pivotal role in the maintenance of cellular homoeostasis in the heart 24, 25. At basal level, autophagy helps to maintain housekeeping functions including cardiomyocyte function and ventricular mass. However, increased autophagy in hypertrophied hearts may play a role in the transition from cardiac hypertrophy to heart failure 21, 26. Autophagy has recently gained much attention as a main route of cell death in the setting of heart failure 27, 28. Interestingly, autophagic activity is commonly increased in the heart under conditions where the RAS is up‐regulated (*e.g*. hypertension, chronic ischaemia, heart failure) 29, 30, 31. It has been reported that Ang II triggers cardiomyocyte autophagy 6. Ang‐(1‐7), the main component of the ACE2–Ang‐(1‐7)–Mas axis, has been shown to effectively oppose the deleterious effects of Ang II 32, 33, 34, 35. Nonetheless, the role of Ang‐(1‐7) in Ang II‐induced cardiomyocyte autophagy and myocardial remodelling remains largely elusive. Data from our current study revealed that Ang‐(1‐7) effectively opposed Ang II‐elicited cardiomyocyte autophagy *via* a Mas receptor‐dependent manner.

Findings from our *in vitro* experiments revealed that Ang II triggered autophagy in cultured cardiomyocytes, the effect of which was alleviated by Ang‐(1‐7). Our data further revealed that Ang‐(1‐7) retarded the Ang II‐induced cardiomyocyte autophagy and cardiac remodelling *in vivo*. Our results showed that Ang‐(1‐7) not only decreased the Ang II‐induced cardiomyocyte autophagy but also reduced cardiac remodelling along with improved cardiac function. These findings indicated that Ang‐(1‐7) is capable of offsetting Ang II‐induced cardiomyocyte autophagy and cardiac remodelling.

The effects of Ang‐(1‐7) are believed to be mediated through the G protein‐coupled receptor Mas which is highly expressed in several tissues including heart, kidney and vasculature 36, 37, 38. To test if Ang‐(1‐7) exerts its actions through Mas receptor, the selective Mas receptor antagonist A779 was employed. Our data indicated that antagonism of the Mas receptor using the peptide inhibitor mitigated the beneficial effects of Ang‐(1‐7) on Ang II‐induced cardiac autophagy and remodelling. These findings supported the notion that Ang‐(1‐7) suppresses Ang II‐induced cardiac autophagy and remodelling through a Mas receptor‐depend mechanism. An earlier study showed that angiotensin (1‐7) induces MAS receptor internalization 39. Further scrutiny is warranted to elucidate whether MAS receptor internalization contribute to the protective effect of Ang‐(1‐7) against Ang II‐induced excessive autophagy and cardiac remodelling.

Oxidative stress has been shown to play a crucial role in Ang II‐induced autophagy 40. The role of oxidative stress as an upstream trigger of autophagy has been characterized. To this end, our study also determined whether Mas receptor mediates the beneficial effect of Ang‐(1‐7) against Ang II‐induced cardiomyocyte autophagy through the inhibition of oxidative stress. Our data revealed that Ang‐(1‐7) effectively inhibited Ang II‐induced oxidative stress and autophagy.

In summary, our study reported for the first time direct beneficial effects of Ang‐(1‐7) on Ang II‐induced autophagy in cardiomyocytes. Our results demonstrated that Mas receptor mediates Ang‐(1‐7)‐offered protective effect against Ang II‐induced cardiomyocyte autophagy and cardiac remodelling through the inhibition of oxidative stress. These findings support the therapeutic utility of targeting the ACE2/Ang‐(1‐7)/Mas receptor axis to reduce cardiomyocytes autophagy. These findings indicate that treatment with Ang‐(1‐7) may be useful to prevent Ang II‐induced excessive autophagy and cardiac remodelling in the heart. In the clinical setting, there is a growing need for novel drugs to prevent or reverse excessive autophagy and cardiac remodelling.

## Funding

National Natural Science Foundation of China (81100145; 81300082; 81570237; 81370390; 81470467), Chinese Medical Doctor Association (DFCMDA201423).

## Conflicts of interest

The authors confirm that there are no conflicts of interest.
